# Does the Addition of Manual Therapy Approach to a Cervical Exercise Program Improve Clinical Outcomes for Patients with Chronic Neck Pain in Short- and Mid-Term? A Randomized Controlled Trial

**DOI:** 10.3390/ijerph17186601

**Published:** 2020-09-10

**Authors:** Jacobo Rodríguez-Sanz, Miguel Malo-Urriés, Jaime Corral-de-Toro, Carlos López-de-Celis, María Orosia Lucha-López, José Miguel Tricás-Moreno, Ana I Lorente, César Hidalgo-García

**Affiliations:** 1 Faculty of Medicine and Health Sciences, Universitat Internacional de Catalunya, 08195 Sant Cugat del Vallès, Spain; carlesldc@uic.es; 2 Faculty of Health Sciences, Universidad de Zaragoza, 50009 Zaragoza, Spain; malom@unizar.es (M.M.-U.); jaimecorral.fisio@gmail.com (J.C.-d.-T.); orolucha@unizar.es (M.O.L.-L.); jmtricas@unizar.es (J.M.T.-M.); hidalgo@unizar.es (C.H.-G.); 3 Physiotherapy Research Unit, Universidad de Zaragoza, 50009 Zaragoza, Spain; 4 Fundació Institut Universitari per a la recerca a l’Atenció Primària de Salut Jordi Gol i Gurina, 08007 Barcelona, Spain; 5 Impact Laboratory, Aragón Institute of Engineering Research (I3A), Universidad de Zaragoza, 50018 Zaragoza, Spain; analorente@unizar.es

**Keywords:** upper cervical spine, manual therapy, training, neck pain

## Abstract

Chronic neck pain is one of today’s most prevalent pathologies. The International Classification of Diseases categorizes four subgroups based on patients’ associated symptoms. However, this classification does not encompass upper cervical spine dysfunction. The aim is to compare the short- and mid-term effectiveness of adding a manual therapy approach to a cervical exercise protocol in patients with chronic neck pain and upper cervical spine dysfunction. Fifty-eight subjects with chronic neck pain and upper cervical spine dysfunction were recruited (29 = Manual therapy + Exercise; 29 = Exercise). Each group received four 20-min sessions, one per week during four consecutive weeks, and a home exercise regime. Upper flexion and flexion-rotation test range of motion, neck disability index, craniocervical flexion test, visual analogue scale, pressure pain threshold, global rating of change scale, and adherence to self-treatment were assessed at the beginning, end of the intervention and at 3- and 6-month follow-ups. The Manual therapy + Exercise group statistically improved short- and medium-term in all variables compared to the Exercise group. Four 20-min sessions of Manual therapy + Exercise along with a home-exercise program is more effective in the short- to mid-term than an exercise protocol and a home-exercise program for patients with chronic neck pain and upper cervical dysfunction.

## 1. Introduction

Chronic neck pain is one of the most prevalent pathologies nowadays, accounting for 14.6% of all musculoskeletal health problems [1]. It is estimated that 50% of all adults experience some kind of neck pain at some point each year [2].

Cervical exercise has been shown to be an effective treatment for neck pain [3,4]. A recent systematic review in patients with chronic neck pain concluded that multimodal training (exercises involving deep and superficial cervical muscles) is necessary to have beneficial effects on function and symptoms [5]. Some studies have considered if a manual therapy approach should be added or not to the cervical exercise protocol for neck pain [4,6]. These studies have been carried out in neck pain subgroups according to the International Classification of Diseases (neck pain with mobility deficits in the global cervical spine, neck pain with radiating pain “radicular pain”, neck pain with movement coordination impairments, and neck pain with cervicogenic headache) and the results are prone to consider the effectiveness of cervical exercise [4,7]. However, there is a lack of clinical trials evaluating the effectiveness of the manual therapy approach on neck pain samples with upper cervical joint dysfunctions. Moreover, more than 60% of cervical axial rotation occurs in the upper cervical spine [8], a fundamental region for cervical function. Lack of mobility and symptoms arising from upper cervical joints are considered to be the main indication for upper cervical manual therapy approach. In addition, upper cervical dysfunction could limit the efficacy of cervical exercise in this sample of chronic neck pain patients. The effect of these treatments in a new subgroup of patients with chronic neck pain and upper cervical spine dysfunction is currently unknown [4,9,10].

The hypothesis of this study is that adding an upper cervical spine manual therapy approach to a cervical exercise protocol is more effective in improving function and symptomatology than an exercise protocol in patients with chronic neck pain and upper cervical spine dysfunction.

The objective of this study aimed to compare the short- and mid-term effectiveness of adding a manual therapy approach to a cervical exercise protocol in patients with chronic neck pain and upper cervical spine dysfunction.

## 2. Methods

### 2.1. Study Design

A randomized, longitudinal (simple 1:1) controlled clinical trial was conducted using the Microsoft Excel program for the randomization by an external researcher. Consolidated Standards of Reporting Trial (CONSORT) guidelines were followed throughout the study. Assignments were placed in a concealed opaque envelope, and participants were randomly assigned to intervention groups. The design was carried out in collaboration with the University of Zaragoza, “Delicias Sur” Health Center of Zaragoza, and the OMT-E Clinical Center of Zaragoza, Spain (Clinicaltrials.gov number: NCT03670719; date of first registration 13 September 2018). This study was approved by the local ethics committee (Comité Ético de Investigación Clínica de Aragón “CEICA”; 13/2018). All research was performed in accordance with relevant guidelines/regulations, and informed consent was obtained from all participants. The subjects in the images in this manuscript gave consent for publication in an online open-access publication.

### 2.2. Sample Size Calculation

The sample size was calculated based on the outcomes of two studies, Dunning et al. 2012 [11] and Izquierdo-Pérez et al. 2014 [12]. The common standard deviation and the minimum differences to be detected between the groups were determined using the outcomes of these two previously mentioned studies [11,12]. The main variables used for sample size calculation of our study were the flexion-rotation test [11] and neck disability index [12], obtaining the highest number of subjects (26 subjects per group using neck disability index variable), making a total sample of at least 52 subjects. The sample size was calculated using the GRANMO 7.12 program, with a α risk of 0.05, test two-side, a β risk of 0.20. For the neck disability index variable, we used an estimated common standard deviation of 6.8 [12] and a minimum expected difference of 5.8 [12], estimating a follow-up loss of 15%.

### 2.3. Subjects

Fifty-eight volunteer subjects were recruited (17 men; 41 women), six patients more than the required sample size. This was the total number of patients referred by doctors over a four-week period. The inclusion criteria comprised: a medical diagnosis of chronic neck pain that persists for more than 3 months [6], a positive result in the flexion-rotation test (less than 32° or an asymmetry of 10° or more between sides) [13,14], a failure to pass stage 2 (24 mmHg) of the craniocervical flexion test [15], hypomobility in one or more segments of C0-1, C1-2, C2-3 through manual assessment according to Zito et al. 2006 [16] and Kaltenborn (2012) [17], grade I-II in the classification of cervical pain [18], being over 18 years old, and having signed the informed consent. Exclusion criteria comprised: contraindications for manual therapy or exercise, having participated in a cervical exercise or manual therapy program in the last three months, presenting warning signs or having suffered a relevant neck trauma [19], an inability to maintain supine position, the use of pacemakers, an inability to perform a flexion-rotation test, language difficulties, and pending litigation or lawsuits [20].

### 2.4. Measurements

The primary outcome measures in this study were the neck disability index and flexion-rotation test. Secondary outcome measures were upper cervical flexion range of motion, pain intensity, craniocervical flexion test, cervical pressure pain threshold, global rating of change scale (GROC-Scale), and adherence to self-treatment scale.

Neck disability was measured using the neck disability index. The test–retest reliability of this questionnaire is excellent (ICC 0.97) and has been validated in the Spanish language [21].

Pain intensity was assessed on a visual analogue scale from 0 to 10 cm in length, with no intermediate point. Test–retest reliability is excellent (ICC 0.92) [22].

A Flexion-rotation test was used to measure the upper rotation, predominantly at C1-2. The methodology proposed by Hall et al. 2007 [13] was followed. The subject was in the supine position, and the evaluator passively moved the patient’s cervical spine to its maximum flexion and then rotated the head to the right and left side with the occiput resting against the evaluator’s abdomen. The movement stopped at whichever situation occurred first, either the subject presented symptoms, or a firm end feel was encountered [13,23]. A CROM device (floating compass; Plastimo Airguide, Inc, Buffalo Groove, IL, USA) was used, and three measurements were taken for each rotation, with the result being the mean of the three measurements [20]. The range of motion to the more restricted (+) and less restricted (-) rotation was considered. Flexion-rotation test reliability is between 0.93–0.96 [24,25] and has excellent validity [26].

Active mobility of upper cervical spine flexion was measured in the standing position using a CROM device [27].

The craniocervical flexion test was used to measure the isolated activation of the deep flexor muscles. A Stabilizer Pressure Biofeedback Unit (Chattanooga, TN, USA) was used to measure this test. The activation and resistance of the deep cervical flexors were evaluated in five progressive pressure increases of 2 mmHg up to a maximum of 30 mmHg. When the patient reached a level three times, he or she passed to the next level [28]. Test–retest reliability is excellent 0.98 ICC [29].

The cervical pressure pain threshold was measured using a digital algometer (Somedic AB Farsta, Somedic SenseLab AB, Sösdala, Sweden) with a round surface area of 1 cm^2^. Pressure was applied at a speed of 1 kg/cm^2^/s perpendicular to the skin. With the subject supine, the pressure pain threshold was assessed over three points bilaterally: first metacarpal joint, C2-3 zygapophyseal joint, and suboccipital muscles. Patients were instructed to press the button of the digital algometer at the exact moment the sensation of pressure changed to pain. The mean of three trials was calculated over each point and used for analysis. Pressure pain threshold measurements have a high reliability (ICC = 0.92–0.99) [23,30,31].

The GROC-Scale was used to measure the personal self-perceived improvement that the patient had experienced [32,33]. The GROC-Scale is considered to be an efficient way to score patients’ perceived clinical change [34]. The test–retest reliability of the GROC-Scale is excellent (ICC = 0.90) [35].

A scale of adherence to self-treatment was designed to discover the frequency that patients did their exercises at home. Patients were asked to choose among the following answers: “I have done the exercises every day, I have done the exercises 4–6 days a week, I have done the exercises 1–3 days a week, I have done the exercises less than 1 day a week, or I have not done them”.

One clinical researcher performed all the treatment for all patients and another clinical researcher, with training in evaluation and more than 5 years’ clinical experience, took all the clinical measurements for all patients before (T0), at the end of the intervention (T1), after 3 months (T2) and after 6 months (T3). This researcher remained blinded to each patient’s assignment group throughout the process. Study participation was then complete, and researchers proceeded with an individualized treatment approach.

### 2.5. Intervention

The intervention was administered individually in the facilities of the Universidad de Zaragoza. Participants in both groups received one 20-min session once a week for four consecutive weeks. The treatment was applied by a researcher with more than 5 years’ experience in physical therapy. A weekly video call was made for all patients to monitor their adherence to these recommendations during the home exercise program. This home exercise program was performed throughout the study.

### 2.6. Exercise Group

**Figure 1:**
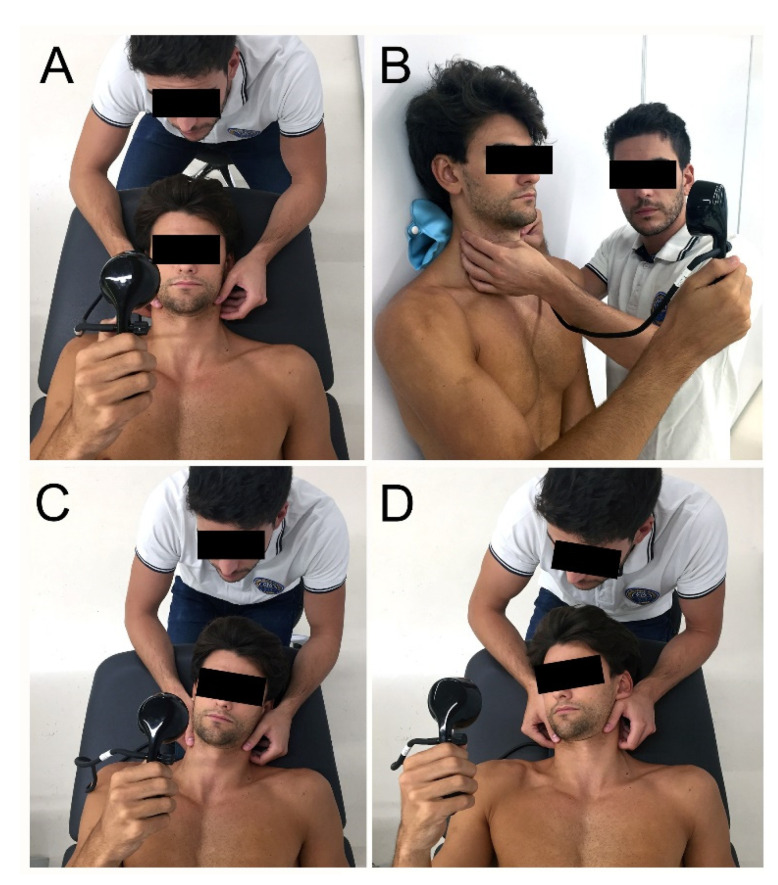
Contraction of deep neck flexor muscle progression. (**A**) Bilateral contraction in supine; (**B**) Bilateral contraction in standing; (**C**) Unilateral contraction with cervical inclination in supine; (**D**) Unilateral contraction with cervical rotation in supine.

The exercise program was developed according to Fernández-de-las-Peñas et al. 2013 [36] and Jull et al. 2002 [37]. This exercise progression includes contraction of deep neck flexor muscles (Figure 1) and global muscles of the neck.

An exercise program was carried out for one day a week for four weeks. Each exercise session lasted 20 min and was composed of two blocks of 10 repetitions, holding each exercise for 10 s, with a 40 s rest between each repetition, and two minutes between blocks [36].

**Figure 2:**
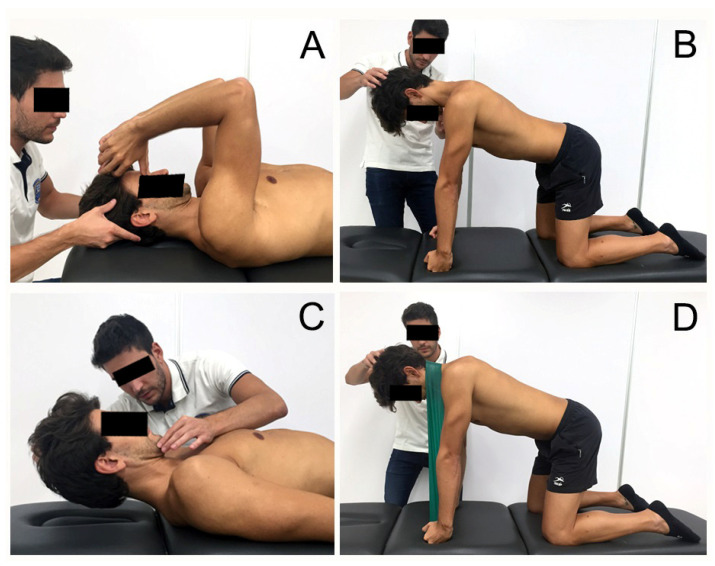
Progression exercises involving superficial muscles. (**A**) Bilateral isometric contraction of superficial and deep flexors muscles; (**B**) Bilateral contraction of deep flexors and extensors in four points; (**C**) Bilateral isometric contraction of superficial and deep flexors muscles against gravity; (**D**) An example of Bilateral contraction of deep flexors and extensors in quadruped with resistance.

After T0 assessments, patients started with the first treatment session performing cervical stabilization exercises and were taught to perform the contraction of deep neck flexor muscle activity with the help of the Stabilizer Pressure Biofeedback Unit (Chattanooga, TN, USA) [28,38]. A progression was also added in the contraction of the deep flexors in different positions in the following sessions (Figure 1). In sessions 2, 3, and 4 with the therapist, exercises involving other muscles and different movements (flexion/extension/rotations/inclinations) were implemented. External resistance was used to increase the intensity of the exercises and was advanced unilaterally towards the most symptomatic side (Figure 2). All exercises were performed with prior contraction of the deep flexors [36]. Moreover, all patients were encouraged to perform home exercises every day between two and five times a day, starting after the first session [37,39,40,41].

### 2.7. Manual Therapy + Exercise Group (MT + E)

**Figure 3:**
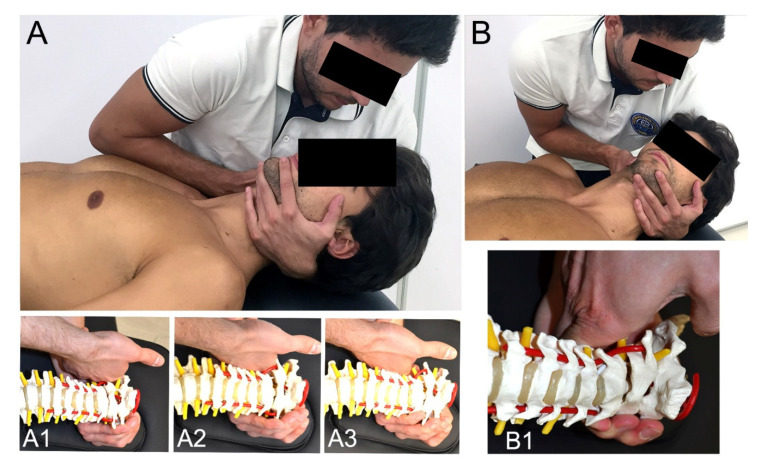
Manipulation Techniques. (**A**) Traction-Manipulation in the Resting Position; (**A1**) Traction-Manipulation in the Resting Position C0-1; (**A2**) Traction-Manipulation in the Resting Position C1-2; (**A3**) Traction-Manipulation in the Resting Position C2-3; (**B,****B1**) Facet Traction-Manipulation C2-3.

**Figure 4:**
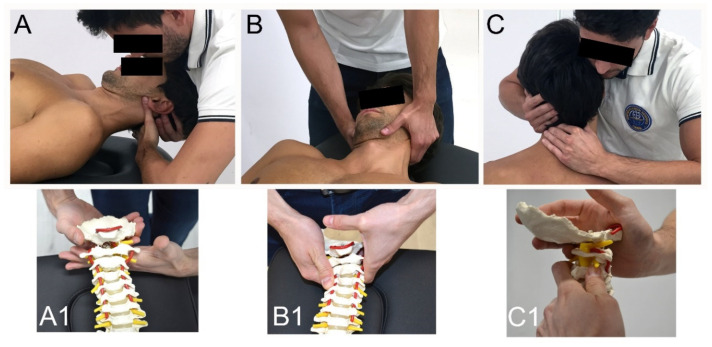
Mobilization Techniques. (**A**,**A1**) Upper Cervical Translatoric Dorsal Glide C0-1; (**B**,**B1**) Ventral-Cranial Glide C2-3 in supine (**C**,**C1**) Upper Cervical Translatoric Dorsal Glide C1-2.

The MT + E program was conducted one day a week for four weeks with the same duration as the Exercise group. Manipulation (high velocity, low amplitude) (Figure 3) and/or mobilization (low velocity, high amplitude) techniques (Figure 4) of the upper cervical spine were combined with cervical exercise [8,17,23,42,43]. The manual therapy techniques used depended on each patient’s clinical findings. The manual therapy approach aimed to restore the mobility of the upper cervical joints by treating occipital-atlas (C0-1) and axis-C3 (C2-3) and then, if necessary, atlas–axis (C1-2) segment. The manipulation and mobilization techniques were always performed with translatoric movements. All the techniques followed the International Federation of Orthopaedic Manipulative Physical Therapists (IFOMPT) recommendations to reduce the risk of adverse effects [19]. The training exercises followed the same progression as the exercise group. The MT + E group rested 30-s between repetitions instead of 40-s during the exercise. This was done to have time to apply the manual therapy techniques and maintain the same session length as the exercise group (20 min).

All patients were encouraged to perform the self-treatment exercises at home every day between two and five times a day after the first session [37,39,40,41].

### 2.8. Statistical Analysis

**Figure 5:**
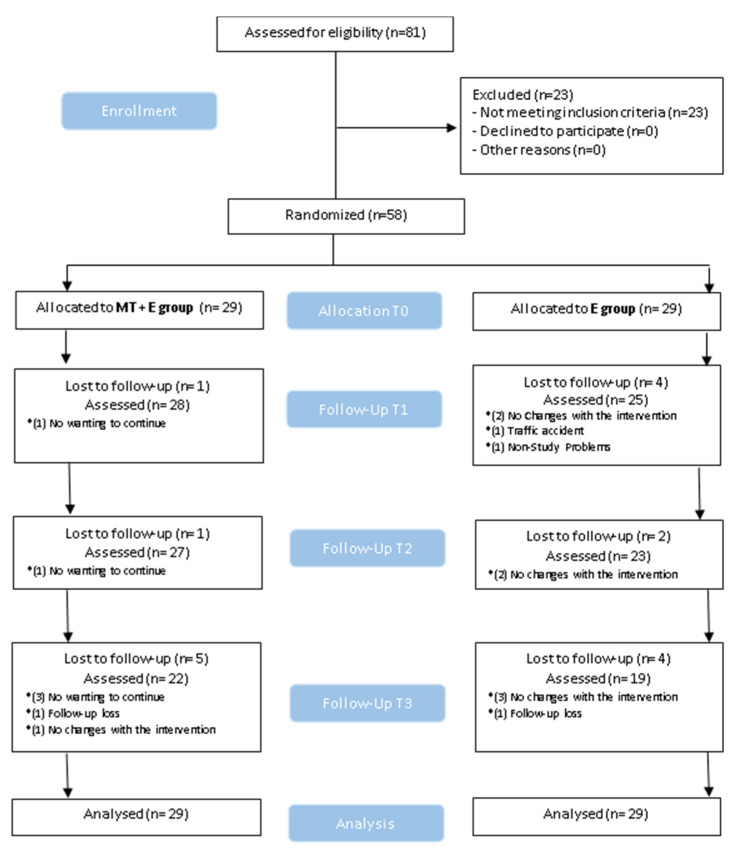
CONSORT. (Consolidated Standards of Reporting Trial) flow diagram.

Statistical analysis was conducted using SPSS 25.0 package (IBM, Armonk, NY, USA). The mean and standard deviations were calculated for each variable. The Kolmogorov–Smirnov test was used to determine a normal distribution of quantitative data (*p* > 0.05). Within- and between-group differences were analyzed using repeated-measures ANOVA and one-way ANOVA for quantitative variables. For qualitative variables, Fisher’s exact test was used. Effect sizes were calculated using Cohen’s d coefficient [44]. An effect size >0.8 was considered large; around 0.5, intermediate; and <0.2, small [44]. Losses and exclusions after randomization are explained in Figure 5. The statistical analysis was performed on an intention-to-treat basis. The level of significance was set at *p* < 0.05.

## 3. Results

Between October 2018 and January 2020, eighty-one volunteers were recruited. Fifty-eight participants (17 men, 41 women) with a mean age of 49.2 (15.9) met all eligibility criteria and agreed to participate. Then 29 participants were randomly assigned to each group, received their assigned treatment, and were analyzed for intention to treat (Figure 5). The demographic characteristics of the sample are summarized in Table 1. Drop-outs, enrollment, exclusions after randomization, and follow-ups are in the flow diagram (Figure 5). There were no adverse events with the treatments performed in the study at any follow-up. However, three exercise group participants reported treatment side-effects, such as mild and transient aggravation of neck pain, in the 6-month follow-up.

### 3.1. End of the Intervention (T1)

**Figure 6:**
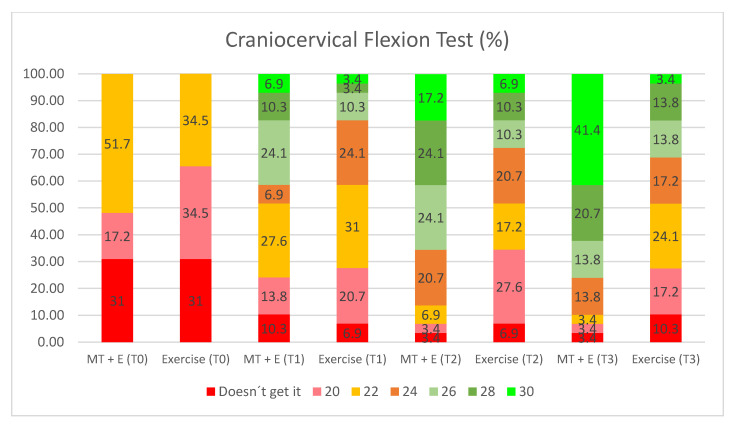
Craniocervical Flexion Test Graphic. Doesn´t get it, the patient is unable to start the test because of pain; 20–30 mmHg, the patient is able to complete the test up to the indicated millimeters of mercury MT + E, Manual Therapy + Exercise.

In the within-group analysis of the exercise group, statistically significant improvement was found in the neck disability index questionnaire and the craniocervical flexion test (*p* < 0.01) (Figure 6). In the MT + E, significant improvement was found in the following variables: visual analogue scale, neck disability index, flexion-rotation test to the more restricted (+) and less restricted (−) side (*p* < 0.01) (Table 2), pressure pain threshold in the suboccipital (right), C2-3 (left), and suboccipital (left) (*p* < 0.05) and craniocervical flexion test (Figure 6).

**Figure 7:**
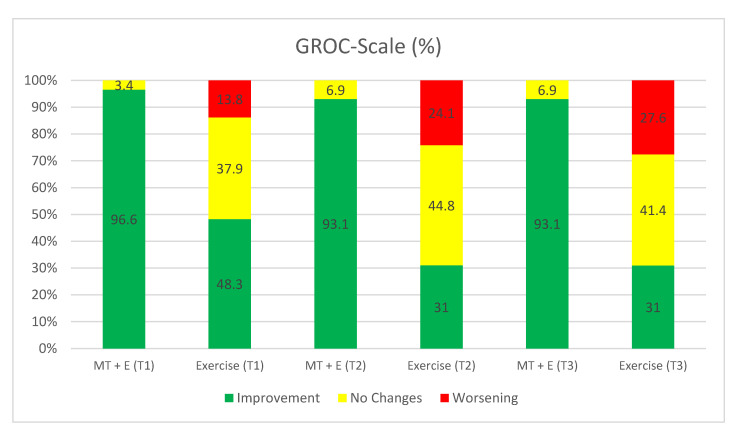
Global rating of change scale (GROC-Scale) Graphic. Patients’ subjective perceptions of clinical change. MT + E, Manual Therapy + Exercise.

In the between-group analysis (Table 3), statistically significant differences were found in favor of the MT + E group in the visual analogue scale, neck disability index, flexion-rotation test to the more restricted (+) and less restricted (−) side (*p* < 0.05) and in the pressure pain threshold variables in C2-3 (right), suboccipital (right), first metacarpal joint (left), C2-3 (left), and suboccipital (left) (*p* < 0.05). Differences in the GROC-Scale variable (*p* < 0.01) (Figure 7) were also identified.

The home exercise regime was performed every day (55.2% MT + E and 65.5% Exercise of participants), 4–6 days a week (31% MT + E and 24.1% Exercise), and 1–3 days a week (13.8% MT + E and 10.3% Exercise). There were no differences between groups (*p* > 0.05).

### 3.2. 3-Month Follow Up (T2)

In the within-group analysis (Table 2) of the exercise group, a statistically significant improvement was found in the craniocervical flexion test variable (*p* < 0.01). However, a statistically significant worsening in the flexion-rotation test to the less restricted side was found (*p* < 0.01). In the MT + E group, there was a statistically significant improvement in the visual analogue scale, upper cervical flexion, neck disability index, flexion-rotation test to the more (+) and less (−) restricted side (*p* < 0.05) and craniocervical flexion test (*p* < 0.01) (Figure 6). There was also a statistically significant improvement in the pressure pain threshold variables at C2-3 (right), suboccipital (right), C2-3 (left), and suboccipital (left) (*p* < 0.05).

In the between-group analysis (Table 3), statistically significant differences were found between both groups in favor of the MT + E group for the visual analogue scale, upper cervical flexion, neck disability index (*p* < 0.05), flexion-rotation test variables to the more (+) and less (−) restricted side (*p* < 0.01) and in the pressure pain threshold variables (first metacarpal joint (right), C2-3 (right), suboccipital (right), first metacarpal joint (left), C2-3 (left) and suboccipital variables (left)) (*p* < 0.05). Differences were also found in the GROC-Scale variable (*p* < 0.01) (Figure 7).

The home exercise regime was performed every day (31% MT + E and 48.3% Exercise of participants), 4–6 days a week (20.7% MT + E and 34.5% Exercise), 1–3 days a week (31% MT + E and 17.2% Exercise), and less than 1 day a week (17.2% MT + E, and 0% Exercise). Significant differences were found between groups in favor of the exercise group (*p* < 0.05).

### 3.3. 6-Month Follow Up (T3)

In the within-group analysis (Table 2) of the exercise group, a statistically significant improvement was found in the craniocervical flexion test (*p* < 0.01). However, a statistically significant worsening in the flexion-rotation test to the less (−) restricted side (*p* < 0.05) was observed. In the group MT + E, statistically significant improvements were found in the visual analogue scale, upper cervical flexion, neck disability index, flexion-rotation test to the more (+) and less (−) restricted side, and craniocervical flexion test (*p* < 0.01) (Figure 6). A statistically significant improvement was found in the pressure pain threshold variables in C2-3 (right), suboccipital (right), first metacarpal joint (left), C2-3 (left), and suboccipital (left) (*p* < 0.01).

In the between-group analysis (Table 3), statistically significant differences were found between both groups in favor of the MT + E group in the visual analogue scale, upper cervical flexion, neck disability index, flexion-rotation test to the more (+) and less (−) restricted side (*p* < 0.01), in the pressure pain threshold variables (first metacarpal joint (right), C2-3 (right), suboccipital (right), first metacarpal joint (left), C2-3 (left), and suboccipital variables (left)), GROC-Scale (*p* < 0.01) (Figure 7), and in the craniocervical flexion test variable (*p* < 0.01) (Figure 6).

The home exercise regime was performed every day (10.3% MT + E and 17.2% Exercise of participants), 4–6 days a week (24.1% MT + E and 51.7% Exercise), 1–3 days a week (48.3% MT + E and 31% Exercise, and less than 1 day a week (17.2% MT + E and 0% Exercise). The Exercise group performed the home exercise more regularly with statistical significance (*p* < 0.05).

## 4. Discussion

The objective of this study was to compare the short- and mid-term effectiveness of adding a manual therapy approach to a cervical exercise protocol in patients with chronic neck pain and upper cervical spine dysfunction. A statistically significant improvement in all study variables was found in the group that received manual therapy and exercise over the exercise group in the short- and mid-term.

Both groups showed statistically significant improvements in the neck disability index at the end of the treatment period (T1). Several articles support the use of cervical exercises leading to positive results in patients´ disabilities [6,40,41,45]. However, the improvement in the MT + E group was more than double that of the exercise group in our study and greater than those found in earlier studies [4,12,46]. At T2 and T3, only the MT + E group showed statistically significant improvements and showed a clinically relevant change (5 and 7 points, respectively) [47]. Similarly, patients’ perception of improvement reflects a statistically significant greater improvement in the MT + E group compared to the exercise group using the GROC-Scale. Different studies consider that patient’s perception is an important variable to take into account in a bio–psycho–social model [48,49,50].

Adding a manual therapy approach focused on treating upper cervical joint restriction of movement to a cervical exercise protocol significantly improved upper cervical mobility in the sagittal and transverse planes. The range of motion of the flexion-rotation test significantly improved after 1 month (T1), 3 months (T2), and 6 months (T3). At T2 and T3, upper cervical flexion also increased significantly. The flexion-rotation test measures the upper cervical rotation, mainly in C1-2 segment [26]. The manual therapy approach aimed to restore the mobility of the upper cervical joints by treating occipital-atlas (C0-1) and axis-C3 (C2-3) and then, if necessary, atlas–axis (C1-2) segment. Apart from treating directly C1-2 [8], the application of manual therapy in C0-1 and C2-3 [8,23,51,52] has been shown to improve the flexion rotation test. Our manual therapy approach followed international safety recommendations promoting the indirect treatment of the segment with more dysfunction (in this case, C1-2) and avoiding end-range procedures [19]. Our study suggests that adding manual therapy to a cervical exercise protocol could be beneficial for maintaining an upper cervical range of motion at a mid-term follow-up. Several studies have shown that this improvement is sustained over the long term for one to three years, even when the continuation of home exercises after initial treatment has been inconsistent [37,53]. However, unlike previous studies [8,19,20], the improvement of the upper cervical range of motion was not present in our exercise group. Our specific inclusion criteria may explain this difference as patients with upper cervical spine restriction of movement could experience greater difficulty in improving the upper cervical range of motion while performing the cervical exercise [54].

In contrast, although there was a tendency to improve the performance in the MT + E group at T3, both groups showed a statistically significant improvement in the craniocervical flexion test in short- and mid-term follow-ups. The craniocervical flexion test measures the activation of the deep cervical flexor muscles. There are several studies that demonstrate the same result when applying manual therapy or MT + E techniques [7,55,56] and exercises [41,57]. The rationale behind using manual therapy may be explained by the linear relationship between upper cervical spine range of motion and the contractile capacity of deep cervical musculature [58] and the improved recruitment in deep muscles and less activation in superficial muscles shown by the application a specific cervical mobilization [59].

Our study showed that adding a manual therapy approach focused on treating upper cervical joint restriction of movement to a cervical exercise protocol significantly diminished the visual analogue scale for pain and increased the pressure pain thresholds in all follow-ups. These results regarding pain alleviation are similar to the studies that found improvements in cervical pain in MT + E groups [7,60], although studies with an isolated manual therapy approach [12] and exercise [61] have also shown a reduction in cervical pain. Considering pressure pain threshold variables, previous studies did not identify any difference when applying manual therapy alone [23,62]. Our MT + E was more effective than the exercise group, showing similar results to Celenay et al., 2016 [5], although other authors found no difference between groups [41,46]. Similar to our results, different studies suggest that manual therapy provokes hypoalgesia locally and in regions distant from the area of the segmental treatment [63,64,65,66,67]. The underlying explanation for this hypoalgesia is the activation of segmental inhibitory pathways, spinal cord pathways, or descending inhibitory pathways of the brain stem [68,69].

### Limitations

The study presents some limitations. The results of this study are limited to a sample presenting several inclusion and exclusion criteria, and only one therapist provided the treatment in the current study, which may limit the generalization of the results. The manual therapy approach was adapted to the clinical finding of the upper cervical spine. This clinical approach did not allow the determination of which specific intervention was more effective. In addition, the exercise protocol included a home exercise regime. Although subjects were periodically asked about and supervised for the performance of the home exercises, the methodology presented limitations in controlling the frequency and execution of the home exercises.

Active function testing of the cervical muscles was limited to the deep ventral flexors with the upper cervical flexion active range of motion and the craniocervical flexion test. Therefore, no generalization of the results to other cervical muscles could be stated. Another limitation is that the last follow-up was done after 6 months, so we cannot know the long-term evolution. Finally, even though patients were referred by doctors, the presence of neck pain with a visceral origin may have been underestimated, so our clinical trial may have taken into account visceral referred neck pain in the inclusion and exclusion criteria [70].

## 5. Conclusions

Adding manual therapy within a program of four 20-min sessions of exercise and home-exercise was found to be more effective than the same exercise program without including manual therapy, in patients with chronic neck pain and upper rotation restriction. Outcomes in neck disability index, patients´ perception of improvement, upper cervical range of motion, pain intensity, and pressure pain thresholds improved in the short- (3-months) and mid-term (6-months) and craniocervical flexion test in the mid-term were better in patients who received manual therapy.

## Figures and Tables

**Table 1:** Baseline features for both groups.

Clinical Features	E Group (*n* = 29)	MT + E Group (*n* = 29)
Age (years)	49.72 ± 17.56	48.76 ± 14.53
Sex	7 M; 22 F	10 M; 19 F
Duration of Symptoms (months)	124.38 ± 148.17	96.97 ± 96.73
Visual Analogue Scale (centimeters)	3.76 ± 2.53	3.36 ± 1.97
Upper Cervical Flexion (°)	10.59 ± 4.39	11.45 ± 4.24
Neck Disability Index	15.24 ± 6.99	12.55 ± 6.25
Doesn’t get it	51.70%	34.50%
20 mmHg	17.20%	34.50%
22 mmHg	31%	31%
Flexion-rotation test (°)		
(+)	12.80 ± 6.04	17.26 ± 7.90
(-)	22.91 ± 10.52	27.12 ± 9.19
Pressure Pain Threshold (kPa)		
First MCJ (R)	359.14 ± 175.98	395.93 ± 195.23
C2-3 (R)	173.76 ± 87.92	208.69 ± 114.53
Suboccipital (R)	186.10 ± 75.34	211.45 ± 91.57
First MCJ (L)	364.34 ± 155.47	339.90 ± 184.74
C2-3 (L)	174.59 ± 90.02	206.38 ± 113.72
Suboccipital (L)	180.59 ± 79.85	207.90 ± 105.33

M, male; F, female; Doesn’t get it, the patient is unable to start the test because of pain; 20–22 mmHg, the patient is able to complete the test up to the indicated millimeters of mercury +, most restricted or less range of movement between the two test rotations; -, less restricted or higher range of movement between the two test rotations; ROM, range of motion; R, right; L, left; MCJ, metacarpal joint; E, Exercise; MT + E, Manual Therapy + Exercise.

**Table 2:** Outcomes variable values within-group.

Group	Variable	T0	T1					T2					T3				
		Baseline	1 Month	Difference between Baseline				3 Months	Difference between Baseline				6 Months	Difference between Baseline			
		Mean ± SD	Mean ± SD	Mean ± SD	F	*p*-Value*^RA^*	d	Mean ± SD	Mean ± SD	F	*p-*Value*^RA^*	d	Mean ± SD	Mean ± SD	F	*p*-Value*^RA^*	d
Exercise Group	Visual Analogue Scale (centimeters)	3.76 ± 2.53	2.89 ± 2.44	−0.87 ± 0.09	2.89	>0.661	0.35	3.87 ± 2.71	0.11 ± 0.18	2.89	>1.000	0.04	3.91 ± 2.84	0.15 ± 0.31	2.89	>1.000	0.06
	Upper Cervical Flexion (°)	10.59 ± 4.39	10.59 ± 5.21	0.00 ± 0.82	2.57	>1.000	0.00	9.03 ± 5.27	−1.56 ± 0.88	2.57	>0.406	0.32	8.93 ± 4.76	−1.66 ± 0.37	2.57	>0.177	0.36
	Neck Disability Index	15.24 ± 6.99	11.03 ± 6.74	−4.21 ± 0.25	6.39	<0.001	0.61	12.83 ± 8.09	−2.41 ± 1.10	6.39	>0.116	0.32	13.10 ± 8.58	−2.14 ± 1.59	6.39	>0.420	0.27
	Flexion-rotation test + (°)	12.80 ± 6.04	15.48 ± 10.34	2.68 ± 4.30	2.27	>0.489	0.32	12.76 ± 7.77	−0.04 ± 1.73	2.27	>1.000	0.01	12.83 ± 9.10	0.03 ± 3.06	2.27	>1.000	0.00
	Flexion-rotation test-(°)	22.91 ± 10.52	23.59 ± 11.21	0.68 ± 0.69	8.52	>1.000	0.06	17.21 ± 8.54	−5.70 ± 1.98	8.52	<0.002	0.60	18.03 ± 10.42	−4.88 ± 0.10	8.52	<0.024	0.47
	Pressure Pain Threshold (kpa)																
	First MCJ (R)	359.14 ± 175.98	351.24 ± 168.84	−7.90 ± 7.14	2.24	>1.000	0.05	310.21 ± 133.37	−48.93 ± 42.61	2.24	>0.145	0.31	304.66 ± 121.12	−54.48 ± 54.86	2.24	>0.198	0.36
	C2-3 (R)	173.76 ± 87.92	166.48 ± 78.91	−7.28 ± 9.01	2.06	>1.000	0.09	149.76 ± 77.08	−24.00 ± 10.84	2.06	>0.542	0.29	145.93 ± 65.41	−27.83 ± 22.51	2.06	>0.258	0.36
	Suboccipital (R)	186.10 ± 75.34	180.79 ± 81.65	−5.31 ± 6.31	0.97	>1.000	0.07	161.59 ± 79.99	−24.51 ± 4.65	0.97	>0.722	0.32	163.97 ± 76.99	−22.13 ± 1.65	0.97	>0.943	0.29
	First MCJ (L)	364.34 ± 155.47	343.45 ± 177.71	−20.89 ± 22.24	1.35	>1.000	0.13	339.93 ± 171.41	−24.41 ± 15.94	1.35	>1.000	0.15	323.41 ± 148.53	−40.93 ± 6.94	1.35	>0.289	0.27
	C2-3 (L)	174.59 ± 90.02	185.90 ± 80.34	11.31 ± 9.68	1.91	>1.000	0.13	159.97 ± 80.15	−14.62 ± 9.87	1.91	>1.000	0.17	168.76 ± 76.65	−5.83 ± 13.37	1.91	>1.000	0.07
	Suboccipital (L)	180.59 ± 79.85	196.90 ± 79.97	16.31 ± 0.08	0.61	>1.000	0.20	181.90 ± 67.35	1.31 ± 12.50	0.61	>1.000	0.02	182.45 ± 65.61	1.86 ± 14.24	0.61	>1.000	0.03
Manual Therapy + Exercise Group	Visual Analogue Scale (centimeters)	3.36 ± 1.97	0.75 ± 1.42	−2.61 ± 0.55	13.91	<0.001	1.52	0.80 ± 1.30	−2.56 ± 1.42	13.91	<0.001	1.53	0.98 ± 1.49	−2.38 ± 0.48	13.91	<0.001	1.36
	Upper Cervical Flexion (°)	11.45 ± 4.24	13.55 ± 4.13	2.10 ± 0.11	10.84	>0.074	0.50	14.83 ± 4.63	3.38 ± 0.39	10.84	<0.022	0.76	16.90 ± 4.83	5.45 ± 0.59	10.84	<0.001	1.20
	Neck Disability Index	12.55 ± 6.25	5.45 ± 5.53	−7.10 ± 0.72	19.26	<0.001	1.20	4.66 ± 5.62	−7.89 ± 0.63	19.26	<0.001	1.33	4.76 ± 5.96	−7.79 ± 0.29	19.26	<0.001	1.28
	Flexion-rotation test + (°)	17.26 ± 7.90	37.79 ± 10.48	20.53 ± 2.58	42.51	<0.001	2.21	35.83 ± 9.61	18.57 ± 1.71	42.51	<0.001	2.25	34.48 ± 12.04	17.22 ± 4.14	42.51	<0.001	1.69
	Flexion-rotation test-(°)	27.12 ± 9.19	41.97 ± 9.47	14.85 ± 0.28	31.50	<0.001	1.59	40.07 ± 8.16	12.95 ± 1.03	31.50	<0.001	1.49	38.66 ± 9.42	11.54 ± 0.23	31.50	<0.001	1.24
	Pressure Pain Threshold (Kpa)																
	First MCJ (R)	395.93 ± 195.23	417.14 ± 194.94	21.21 ± 0.29	3.72	>1.000	0.11	431.00 ± 193.54	35.07 ± 1.69	3.72	>1.000	0.18	479.00 ± 214.95	83.07 ± 19.72	3.72	>0.182	0.41
	C2-3 (R)	208.69 ± 114.53	250.83 ± 113.26	42.14 ± 1.27	4.56	>0.108	0.33	277.86 ± 135.35	19.17 ± 20.82	4.56	<0.016	0.49	305.83 ± 162.70	97.14 ± 48.17	4.56	<0.004	0.69
	Suboccipital (R)	211.45 ± 91.57	257.55 ± 112.31	46.10 ± 20.74	10.35	<0.016	0.45	297.72 ± 117.74	86.27 ± 26.17	10.35	<0.001	0.82	344.48 ± 171.01	133.03 ± 79.44	10.35	<0.001	0.97
	First MCJ (L)	339.90 ± 184.74	397.97 ± 172.87	58.07 ± 11.87	6.12	>0.387	0.33	421.72 ± 178.22	81.82 ± 6.52	6.12	>0.120	0.45	483.52 ± 198.00	143.62 ± 13.26	6.12	<0.003	0.75
	C2-3 (L)	206.38 ± 113.72	279.76 ± 167.66	73.38 ± 53.94	10.19	<0.004	0.51	298.14 ± 161.58	91.76 ± 47.86	10.19	<0.001	0.66	332.52 ± 166.91	126.14 ± 53.19	10.19	<0.001	0.88
	Suboccipital (L)	207.90 ± 105.33	267.00 ± 116.26	59.10 ± 10.93	15.22	<0.002	0.53	314.14 ± 155.73	106.24 ± 50.40	15.22	<0.001	0.80	380.66 ± 198.09	172.76 ± 92.76	15.22	<0.001	1.09

Abreviature: SD, Standard Deviation; +, most restricted or less range of movement between the two test rotations; −, less restricted or higher range of movement between the two test rotations; MCJ, First Metacarpal Joint; R, Right; L, Left; *^RA^.* Repeated measures ANOVA; d. Cohen’s d coefficient.

**Table 3:** Outcomes variable values between-group.

Variable	Group	T0	T1					T2					T3				
		Baseline	1 Month	Difference between Groups				3 Months	Difference between Groups				6 Months	Difference between Groups			
		Mean ± SD	Mean ± SD	Mean ± SD	F	*p-*Value *^OA^*	d	Mean ± SD	Mean ± SD	F	*p-*Value *^OA^*	d	Mean ± SD	Mean ± SD	F	*p*-Value *^OA^*	d
Visual Analogue Scale (centimeters)	E Group	3.76 ± 2.53	2.89 ± 2.44	2.14 ± 1.02	6.23	<0.016	0.78	3.87 ± 2.71	3.07 ± 1.41	15.50	<0.001	1.44	3.91 ± 2.84	2.93 ± 1.35	14.73	<0.001	1.29
	MT + E Group	3.36 ± 1.97	0.75 ± 1.42					0.80 ± 1.30					0.98 ± 1.49				
Upper Cervical Flexion (°)	E Group	10.59 ± 4.39	10.59 ± 5.21	2.96 ± 1.08	3.74	>0.058	0.63	9.03 ± 5.27	5.80 ± 0.64	13.47	<0.001	1.17	8.93 ± 4.76	7.97 ± 0.07	36.33	<0.001	1.66
	MT + E Group	11.45 ± 4.24	13.55 ± 4.13					14.83 ± 4.63					16.90 ± 4.83				
Neck Disability Index	E Group	15.24 ± 6.99	11.03 ± 6.74	5.58 ± 1.21	4.35	<0.042	0.74	12.83 ± 8.09	8.17 ± 2.47	14.78	<0.001	1.17	13.10 ± 8.58	8.34 ± 2.62	12.21	<0.001	1.13
	MT + E Group	12.55 ± 6.25	5.45 ± 5.53					4.66 ± 5.62					4.76 ± 5.96				
Flexion-rotation test + (°)	E Group	12.80 ± 6.04	15.48 ± 10.34	22.31 ± 0.14	53.47	<0.001	2.14	12.76 ± 7.77	23.07 ± 1.84	78.84	<0.001	2.64	12.83 ± 9.10	21.65 ± 2.94	45.55	<0.001	2.03
	MT + E Group	17.26 ± 7.90	37.79 ± 10.48					35.83 ± 9.61					34.48 ± 12.04				
Flexion-rotation test-(°)	E Group	22.91 ± 10.52	23.59 ± 11.21	18.38 ± 1.74	44.37	<0.001	1.77	17.21 ± 8.54	22.86 ± 0.38	70.91	<0.001	2.74	18.03 ± 10.42	20.63 ± 1.00	59.36	<0.001	2.08
	MT + E Group	27.12 ± 9.19	41.97 ± 9.47					40.07 ± 8.16					38.66 ± 9.42				
Pressure Pain Threshold (Kpa)																	
First MCJ (R)	E Group	359.14 ± 175.98	351.24 ± 168.84	65.90 ± 26.10	0.53	>0.471	0.36	310.21 ± 133.37	120.79 ± 60.17	4.14	<0.047	0.73	304.66 ± 121.12	174.34 ± 93.83	9.88	<0.003	1.00
	MT + E Group	395.93 ± 195.23	417.14 ± 194.94					431.00 ± 193.54					479.00 ± 214.95				
C2-3 (R)	E Group	173.76 ± 87.92	166.48 ± 78.91	84.35 ± 34.35	5.74	<0.020	0.86	149.76 ± 77.08	128.10 ± 58.27	13.89	<0.001	1.16	145.93 ± 65.41	159.90 ± 97.29	19.01	<0.001	1.29
	MT + E Group	208.69 ± 114.53	250.83 ± 113.26					277.86 ± 135.35					305.83 ± 162.70				
Suboccipital (R)	E Group	186.10 ± 75.34	180.79 ± 81.65	76.76 ± 30.66	7.35	<0.009	0.78	161.59 ± 79.99	136.13 ± 37.15	25.37	<0.001	1.35	163.97 ± 76.99	180.51 ± 94.02	28.23	<0.001	1.36
	MT + E Group	211.45 ± 91.57	257.55 ± 112.31					297.72 ± 117.74					344.48 ± 171.01				
First MCJ (L)	E Group	364.34 ± 155.47	343.45 ± 177.71	54.52 ± 4.84	4.10	<0.048	0.31	339.93 ± 171.41	81.79 ± 6.81	7.15	<0.010	0.47	323.41 ± 148.53	160.11 ± 49.47	20.24	<0.001	0.92
	MT + E Group	339.90 ± 184.74	397.97 ± 172.87					421.72 ± 178.22					483.52 ± 198.00				
C2-3 (L)	E Group	174.59 ± 90.02	185.90 ± 80.34	93.86 ± 87.32	7.47	<0.008	0.71	159.97 ± 80.15	138.17 ± 81.43	20.91	<0.001	1.08	168.76 ± 76.65	163.76 ± 90.26	26.36	<0.001	1.26
	MT + E Group	206.38 ± 113.72	279.76 ± 167.66					298.14 ± 161.58					332.52 ± 166.91				
Suboccipital (L)	E Group	180.59 ± 79.85	196.90 ± 79.97	70.10 ± 36.29	4.64	<0.036	0.70	181.90 ± 67.35	132.24 ± 88.35	16.92	<0.001	1.10	182.45 ± 65.61	198.21 ± 132.48	28.06	<0.001	1.34
	MT + E Group	207.90 ± 105.33	267.00 ± 116.26					314.14 ± 155.73					380.66 ± 198.09				

Abbreviature: E, Exercise; MT + E, Manual Therapy + Exercise; SD, Standard Deviation; +, most restricted or less range of movement between the two test rotations; −, less restricted or higher range of movement between the two test rotations; MCJ, First Metacarpal Joint; R, Right; L, Left; *^OA^*. One-way ANOVA; d. Cohen’s d coefficient.

## Data Availability

The datasets used and/or analyzed during the current study are available from the corresponding author on reasonable request.
